# Genetics in Familial Intrahepatic Cholestasis: Clinical Patterns and Development of Liver and Biliary Cancers: A Review of the Literature

**DOI:** 10.3390/cancers14143421

**Published:** 2022-07-14

**Authors:** Giovanni Vitale, Alessandro Mattiaccio, Amalia Conti, Laura Turco, Marco Seri, Fabio Piscaglia, Maria Cristina Morelli

**Affiliations:** 1 Internal Medicine Unit for the Treatment of Severe Organ Failure, IRCCS Azienda Ospedaliero-Universitaria di Bologna, 40138 Bologna, Italy; laura.turco@aosp.bo.it (L.T.); mariacristina.morelli@aosp.bo.it (M.C.M.); 2 U.O. Genetica Medica, IRCCS Azienda Ospedaliero-Universitaria di Bologna, 40138 Bologna, Italy; alessandro.mattiaccio@unibo.it (A.M.); amalia.conti@aosp.bo.it (A.C.); marco.seri@unibo.it (M.S.); 3 Department of Medical and Surgical Sciences (DIMEC), Alma Mater Studiorum-University di Bologna, 40138 Bologna, Italy; 4 Division of Internal Medicine, Hepatobiliary and Immunoallergic Diseases, IRCCS Azienda Ospedaliero-Universitaria di Bologna, 40138 Bologna, Italy; fabio.piscaglia@unibo.it

**Keywords:** progressive familial intrahepatic cholestasis, Alagille syndrome, hepatobiliary cancers, hepatocellular carcinoma, cholangiocarcinoma, gallbladder cancer, bile acids, next-generation sequencing, microbiota, liver transplantation

## Abstract

**Simple Summary:**

An increase in serum bile acids can generate a chronic inflammatory state and has been associated with the risk of developing hepatobiliary cancers. Progressive familial intrahepatic cholestasis, other forms of inherited cholestasis, and microbiota dysbiosis lead to an increase in bile acids in the blood, liver and gut. However, mutations in the genes responsible for cholestatic disorders can also be accountable for non-progressive and clinically mild phenotypes. This review summarizes the relationship between inherited cholestasis, bile acids, gut microbiota and the risk occurrence of hepatobiliary tumours.

**Abstract:**

The family of inherited intrahepatic cholestasis includes autosomal recessive cholestatic rare diseases of childhood involved in bile acids secretion or bile transport defects. Specific genetic pathways potentially cause many otherwise unexplained cholestasis or hepatobiliary tumours in a healthy liver. Lately, next-generation sequencing and whole-exome sequencing have improved the diagnostic procedures of familial intrahepatic cholestasis (FIC), as well as the discovery of several genes responsible for FIC. Moreover, mutations in these genes, even in the heterozygous status, may be responsible for cryptogenic cholestasis in both young and adults. Mutations in FIC genes can influence serum and hepatic levels of bile acids. Experimental studies on the *NR1H4* gene have shown that high bile acids concentrations cause excessive production of inflammatory cytokines, resistance to apoptosis, and increased cell regeneration, all risk conditions for developing hepatocellular carcinoma (HCC) and cholangiocarcinoma (CCA). *NR1H4* gene encodes farnesoid X-activated receptor having a pivotal role in bile salts synthesis. Moreover, HCC and CCA can emerge in patients with several FIC genes such as *ABCB11*, *ABCB4* and *TJP2*. Herein, we reviewed the available data on FIC-related hepatobiliary cancers, reporting on genetics to the pathophysiology, the risk factors and the clinical presentation.

## 1. Introduction

Progressive familial intrahepatic cholestasis (PFIC), Alagille syndrome, ductal plaque abnormalities such as Caroli’s syndrome, congenital hepatic fibrosis, metabolic diseases including citrine deficiency, and finally, bile acid synthesis defects belong all to hereditary cholestatic disorders.

PFIC is a group of autosomal recessive cholestatic diseases caused by defects in hepatobiliary transport proteins, affecting especially newborns and children; since PFIC often evolves in liver failure and/or liver cancer, it represents an indication for liver transplantation (LT). In addition, the genes responsible for PFIC can cause other non-progressive biliary disorders: low-phospholipid-associated cholelithiasis (LPAC), benign recurrent intrahepatic cholestasis (BRIC), drug-induced cholestasis (DIC) and intrahepatic cholestasis of pregnancy (ICP) [1].

Bile acids (BAs) are steroid-based molecules synthesized from cholesterol in the liver and stored in the gallbladder. BAs regulate glucose and lipid homeostasis and drug absorption, functioning as molecules able to control energy metabolism and its several trafficking pathways.

Recently, the focus has moved to the enterohepatic circulation of BAs and gut microbiota; the misexpression of specific BAs transporters, combined with dysbiosis and imbalance of some gut microbes, have a crucial role in the modulation of inflammation, immunity and metabolism, finally leading to malignant transformation and development of hepatobiliary cancers (HBCs) [2,3,4].

In the last years, the continuous advances in diagnostic methods as well as next-generation sequencing (NGS), whole-exome sequencing (WES) and whole-genome sequencing (WGS) led to the finding of new genes responsible for cholestatic diseases and a better understanding of the relationship between a broad spectrum of cholestatic disorders and the development of HBCs.

As matter of fact then, several genes involved in the development of inherited cholestasis are linked to the risk of HCC and CCA in pediatric and not pediatric populations: *ABCB11*, *ABCB4*, *TJP2*, *FXR*, *MYO5B*, *SLC51B*, *SLC25A13*, *NOTCH2*, *JAG1*, *TGR5* and *HNF1B* [5,6,7].

Furthermore, mutations in genes responsible for cholestasis may be present in children and adults with non-progressive forms of cryptogenic cholestasis, even in their heterozygous status [1,8].

Primary liver cancer is the sixth most common cancer worldwide, and it typically develops in the setting of cirrhosis. However, 20% of these cases can occur in non-cirrhotic livers [9,10].

Chronic hepatitis B and C, alcoholic liver disease and nonalcoholic steatohepatitis are the most common causes of liver cancer. Still, in some instances, HCC is detected incidentally during routine image examination with no definite aetiology.

Since altered BAs levels and their composition have a crucial role in HBCs development, it becomes obvious to think that sporadic primary liver cancers could be the result of mutations in genes involved in BAs transport and metabolism, especially in patients with no clear liver disease or with cryptogenic cholestasis.

**Figure 1:**
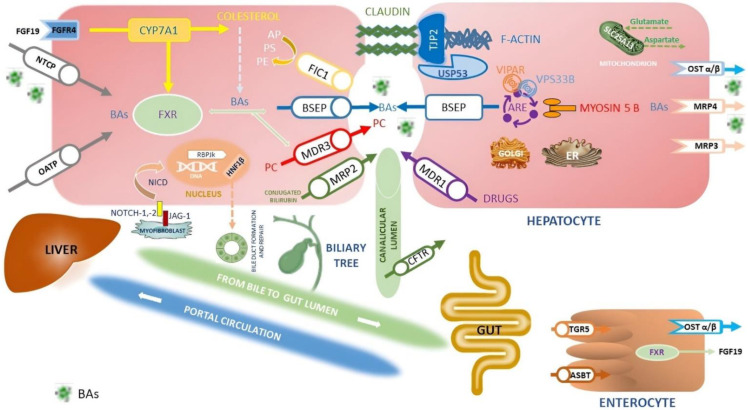
Simplified representation of the proteins expressed by the genes involved in cholestasis disorders and the main pathway of synthesis, transport and reuptake of BA in the liver and in the gut. Here we summarized the primary inherited cholestatic disorders in which metabolic and hepatobiliary diseases cause impaired BAs excretion. BAs are synthesized from cholesterol by CYP7A1 and then transported into canaliculi through the BSEP (PFIC2). Liver storage of BAs leads to liver injury, itching and increased risk of HBCs. Other constituents of bile include PC, transported by canalicular MDR3 (PFIC3), and PS, shuttled by canalicular ATP8B1 (PFIC1). Between disorders of membrane transporter or polarity, there is Dubin Johnson syndrome, where mutations in *ABCC2* cause defects in MRP2, organic anions and bilirubin glucuronide transporter. Instead, *ATP8B1* encodes MDR1 that translocates drugs and phospholipids across the hepatocyte membrane; it is responsible for developing resistance to anticancer drugs. The TJP2 (PFIC4), Claudine (neonatal ichthyosis sclerosing cholangitis), and USP53 (PFIC8) proteins are necessary to maintain the canalicular membrane polarity of hepatocytes and inhibit the reflux of BAs back into the cell: disorders of cytoskeletal and tight junction proteins cause cholestasis. The primary regulator of BAs metabolism is FXR (PFIC5): FXR inhibits CYP7A1 expression, stimulates the synthesis of FGF-19 to inhibit CYP7A1 expression through the FGFR4 pathway in the hepatocytes, stimulates BSEP to export of BAs, downregulates NTCP repressing the uptake of BAs by the liver, finally increases the expression of OST-α/β involved in BAs excretion from the liver to the portal vein and in intestinal reuptake. MYO5B, responsible for a form of PFIC and microvillous inclusion disease, interacts with RAB11A, altering the targeting of BSEP to the canalicular membrane via ARE; mutations in genes encoding RE-associated proteins such as MYO5B, VPS33B, and VIPAR (arthrogryposis, renal dysfunction and cholestasis syndrome—ARC) highlights the role of the RE in establishment and maintenance of hepatocyte polarity. BAs are carried into the hepatocyte by NTCP, OST-α, and OST-β on the basolateral membrane. Instead, ASBT on the ileal enterocyte reabsorbs approximately 95% of BAs, which enter the portal circulation via enterocyte transporters OST-α, OST-β, and MRP3. Mutations in *SLC51A* encoding the OSTα-OSTβ proteins cause PFIC6. Together with FXR, TGR5 is a primary BAS-sensing receptor involved in the interaction between BAs and microbiota; it is a negative regulator of the HCC envelope through its anti-inflammatory properties and abilities to guarantee correct homeostasis of BAs. *SLC25A13* gene encodes the calcium-binding protein citrin, an aspartate–glutamate carrier sited within the inner mitochondrial membrane. Citrin plays a crucial role in protein, nucleotide, and urea synthesis in several metabolic pathways. Mutations in *SLC25A13* lead to neonatal intrahepatic cholestasis caused by citrin deficiency (NICCD) and increased susceptibility to HBCs. Cell–cell Jagged/Notch interactions are critical for the differentiation of cells in the early phases of development. The ligand–receptor link induces proteolytic cleavage of the Notch receptor and release of the NICD. The NICD translocates into the nucleus where it activates RBPJκ, thus promoting Notch target genes’ transcription, including *HNF1B*. The persistent over-activation of Notch way in hepatic precursor cells leads to downstream RBPJk-dependent transcription activity, failing repair cell damage, induction of liver fibrosis and secondary HCC. Mutations in *JAG1* and *NOTCH2* genes are responsible for Alagille syndrome, while *HNF1B* is a target gene upregulated by NOTCH signalling. *HNF1B* regulates the differentiation of hepatoblasts into ductal plate cells and the inclusion of the developing duct into the portal space; mutations in the *HNF1B* gene have been associated with renal cysts and diabetes syndrome neonatal or late-onset cholestasis and some tumours, including liver cancer. CFTR (responsible for cystic fibrosis) is a chloride channel expressed by secretory epithelia, including the biliary epithelium in the liver. Furthermore, mutations affecting the function of CFTR can cause a cholestatic disorder: biliary architecture changes, severe sclerosing cholangitis, focal biliary cirrhosis and multi-lobular biliary cirrhosis complicated by portal hypertension are features of cystic fibrosis liver disease (CFLD). Finally, we omitted KIF12 in the figure since its localization in the Golgi apparatus and plasma membrane is uncertain: mutations in the *KIF12* gene are associated with PFIC8. Abbreviations: ARE; apical recycling endosome; ASBT, apical sodium-dependent bile acid transporter; AP, amino-phospholipids; BAs, bile acids; BSEP, bile salt export pump protein; CFTR, cystic fibrosis transmembrane conductance regulator; CYP7A1, cholesterol 7α-monooxygenase; ER, endoplasmic reticulum; FGF19, fibroblast growth factor 19; FGFR14, fibroblast growth factor receptor 4; FIC 1, familial intrahepatic cholestasis deficiency type 1 protein; FXR, farnesoid X receptor; HNF-1B, Hepatocyte Nuclear Factor-1beta; JAG-2, Jagged Canonical Notch Ligand-2; KIF12, kinesin family member 12; MDR, multidrug resistance protein; MRP, multidrug resistance protein; NICD, Notch intracellular domain; NOTCH-1,2, Notch homolog-1,2 translocation-associated; NTCP, sodium taurocholate cotransporting polypeptide; OATP, organic anion transporting polypeptide; OST α/β, organic solute transporter alpha/beta; PC, phosphatidylcholine; PE, phosphatidylethanolamine; PS, phosphatidylserine; PFIC, progressive familial intrahepatic cholestasis; RBPjk, recombining binding protein suppressor of hairless; SL25A13, solute carrier family 25 member 13; TGR5, G-protein-coupled bile acid receptor; TJP2, tight junction protein 2 gene; USP53, ubiquitin-specific peptidase 53; VIPAR, VPS33B interacting protein, apical–basal polarity regulator; VPS33B, vacuolar protein sorting associated protein 33B [6,7,12,13].

Herein, we reviewed [11] the available data on inherited cholestatic diseases and HBCs, the old and the recently discovered cholestasis-related genes, the underlying pathophysiological mechanisms and finally, the interaction between gene mutations and environmental factors, as well as the enterohepatic circulation of BAs and the gut microbiota (Figure 1 summarizes genes, year of discovery and main phenotypes associated with the cholestatic diseases and the HBCs).

## 2. Materials and Methods

The literature search, carried out by 31 March 2022, included e-Pub published articles (peer-reviewed original articles, reviews and meta-analyses) with the following search terms: “Alagille Syndrome”, “Benign Intrahepatic Cholestasis”, “Citrin deficiency”, “Cholangiocellular carcinoma”, “Familial Intrahepatic Cholestasis”, “Drug-Induced Cholestasis”, Gallbladder Cancer”, “Hepatocellular Carcinoma”, “Hepatobiliary Cancer”, “Intrahepatic Cholestasis of Pregnancy”, “Liver cancer”, “Low-Phospholipid-Associated Cholelithiasis”, “Progressive Familial Intrahepatic Cholestasis”, “ATP8B1”, “ABCB11”, “ABCB4”, “Bile salts”, “Bile acids”, “BSEP”, “CCA”,“HCC”, “HNF1B”, “FIC”, “FGFG19”, “FXR”, “JAG1”, “KIF12”, “MDR3”, “TJP2”, “MYO5B”, “NR1H4”, “NOTCH2”, “PFIC”, “SLC25A13”, “SLC51B”, “TGR5”, “USP53”.

In addition to researching articles published via e-Pubs, we consulted OMIM^®^, Online Mendelian Inheritance in Man^®^, an international resource of human genes and genetic phenotypes (http://omim.org accessed on 31 March 2022), for publications on hereditary cholestasis and HBCs to ensure more excellent research coverage.

We recovered additional articles from references.

Identified articles were manually extracted according to the title and abstract by two expert reviewers in the field.

We cited only studies that provided information:

– On the discovery of cholestasis-related genes;
– On pathological pathways of mutations in these *loci;*
– On epidemiology and clinical features of patients with hereditary cholestatic diseases, focusing on the dysregulation of BAs in the liver gut axis concerning the development of primary biliary and liver cancers.

We applied the following exclusion criteria:

– Abstracts or posters of congresses and meetings;
– Editorials;
– Studies that included patients with intrahepatic cholestasis without a genetic or histological diagnosis;
– Articles focused on patients with other causes of autoimmune or acquired cholestasis, increased BAs such as primary biliary cholangitis, primary sclerosing cholangitis, IgG4 cholangitis, alcoholic and nonalcoholic steatohepatitis.

## 3. From Rare Pediatric Cholestatic Diseases to Adult Cryptogenic Cholestasis: An Overview of Different Features of Familial Intrahepatic Cholestasis

Historically, inherited cholestatic diseases such as PFIC and Alagille syndrome are considered neonatal and pediatric disorders, characterized by a defect in BAs transport or structure of tight junctions at the level of hepatocyte, secondarily impairing bile excretion and liver accumulation resulting in itching, hepatocyte injury and cirrhosis [14]. Eight different PFIC types have now been described, other than *MYO5B*, which is another gene responsible for a clinical picture similar to PFICs.

Figure 1 summarizes the proteins involved in BAs metabolism and transport, their role and their position in hepatocytes, biliocytes and intestinal cells.

The eight genes involved in these mendelian diseases are present on OMIM^®^. Table 1 summarizes genes, year of discovery, and main phenotypes correlated to PFIC including association with the HBCs cases:

– *ATP8B1* gene (PFIC1): it is responsible for the synthesis of a lipid flippase, able to maintain the asymmetry of the cell membrane by the translocation of phospholipids from the exoplasmic to the cytoplasmic leaflet, having a protective role against excessive concentrations of BAs [14];
– *ABCB11* gene (PFIC2): coding for the bile export pump (BSEP), *ABCB11* regulates the excretion of monovalent BAs from hepatocytes to bile canaliculi against a concentration gradient. The accumulation of BAs in hepatocytes is induced by a less expression or a malfunction of BSEP, resulting in cellular injury and alterations of the enterohepatic pathway of BAs [14];
– *ABCB4* gene (PFIC3): alterations of the MDR3 glycoprotein, a phosphatidylcholine flippase sited in the canalicular membrane of hepatocytes, lead to the disease; MDR3 protein carries phosphatidylcholine from the hepatocytes into the bile canaliculus, protecting the cholangiocytes from the detergent activity of BAs and reducing cellular injury. Patients with PFIC3 have, in fact, late onset of disease, and present high levels of gamma-glutamyl transferase (GGT) compared to PFIC1 and PFIC2, in which GGT is low or in the normal range [14];
– *TJP2* gene (PFIC4): *TJP2* encodes an essential protein in the structure of tight junctions, establishing connections between the transmembrane tight junction proteins and the actin cytoskeleton. PFIC4 usually affects pediatric patients with low GGT levels [12];
– *NR1H4* gene (PFIC5): *NR1H4* produces the farnesoid x receptor (FXR), bile acid-activated nuclear hormone receptor, the primary regulator of BAs metabolism and homeostasis [14];
– *SLC51A* (PFIC6): this gene is responsible for the synthesis of the alpha subunit of the alpha-beta heteromeric organic solute transporter (OSTα-OSTβ), having the central role in the intestinal BA reabsorption in the setting of enterohepatic circulation: the pump exports BA across the basolateral membrane and OSTα deficiency causes a pediatric clinical picture with elevated liver transaminases, high GGT-cholestasis, normal serum BAs and congenital diarrhoea [15,16];
– *USP53* (PFIC7): *USP53* encodes a nonprotease homolog of the ubiquitin-specific peptidase family; mutations in this gene are responsible for an autosomal recessive liver disorder characterized by infantile-onset jaundice and itching associated with cholestasis, elevated transaminases, normal GGT, hepatocellular and canalicular cholestasis with fibrotic changes at liver histology. In many cases, resolution of the liver injury is observed with age, although some patients have persistent hepatitis or splenomegaly. A subset of patients develops hearing loss since the *USP53* locus interacts with the tight junction proteins TJP1 and TJP2 in polarized epithelial cells. Mutations in *USP53* alter auditory hair cells modifying the stability of tight junctions [17];
– *KIF12* (PFIC8): mutations in *KIF12* are characterized by cholestasis and high GGT presenting in the infantile period; liver immuno-staining of patients with *KIF12* mutations resulted in changes in MRP2 (*ABCC2* gene) trafficking with its strong cytoplasmic signal leading to change in cell polarity [18].

Mutations in the *MYO5B* gene are associated with the microvillus inclusion disease (MVID), a congenital disorder of the enterocyte that leads to intractable diarrhoea. In addition, intrahepatic pediatric cholestasis can be present since the MYO5B protein interacts with recycling endosome-associated RAB11A protein. This interface is essential for polarized epithelial cells, such as bile canaliculus formation [12].

The same genes responsible for the progressive forms of intrahepatic cholestasis, PFIC, have been involved in other, often non-progressive phenotypes, with clinical implications usually present in adults. The known diseases are:

– Benign recurrent intrahepatic cholestasis (BRIC): BRIC is an inherited disease characterized by almost two episodes of intermittent cholestasis with jaundice. Two types of BRIC are fully known, BRIC1 having mutations in the *ATP8B1* gene and BRIC2 having mutations in the *ABCB11* gene. The clinical presentation is usually less aggressive than PFIC since the protein function is only partially injured; pregnancy, infections, or drugs can trigger the attacks, while liver tests are normal between two episodes [19]. In the last years also, defects in the *MYO5B* gene have been described in patients with recurrent and transient forms of intrahepatic cholestasis [20,21]. Bull et al. reported BRIC-like phenotypes in a patient with *USP53* mutations: age-onset was between infancy and 15 years, and patients had recurrent attacks of cholestasis with low GGT, hyperbilirubinemia and variably increased transaminases [22].
– Intrahepatic cholestasis of pregnancy (ICP): the disease is the most common liver disorder of pregnancy, historically ascribed to the heterozygous mutations in *ATP8B1*, *ABCB11* and *ABCB4* genes. There are also associations with *NR1H4* e *TJP2* genes [23,24]. The main features are transient cholestasis and itching during the pregnancy that resolve after childbirth. Serious fetal complications are rare and occur when levels of BAs are higher than 40 µmol/L with ursodeoxycholic acid (UDCA) as the first-line therapy.

Moreover, women with ICP have an increased risk of later HBCs, immune-mediated and cardiovascular disease [25].

Drug-induced cholestasis (DIC): DIC accounts for 30% of drug-induced liver injury (DILI); DILI is the most frequent cause of acute liver hepatitis globally since many drugs cause cholestasis through the interaction with hepatic transporters.

Indeed, several ATP-dependent canalicular transporters as well as BSEP protein (*ABCB11*), the multidrug resistance protein-2 (MRP2, *ABCC2*), the multidrug resistance-1 protein (MDR1, *ABCB1*) and the MDR3 protein (*ABCB4*) control the complete removal of lipophilic drugs and their metabolites by the liver and the BAs. (Figure 1). The most studied genes are *ABCB11* and *ABCB4*, also responsible for PFIC1 and PFIC2; defects in these hepatobiliary transporter proteins can cause acute or chronic DIC. Many drugs are involved in DIC with an often difficult differential diagnosis [19]. A helpful tool in the diagnosis of DIC is the LiverTox website (www.livertox.nih.gov accessed on 31 March 2022): it is an informative instrument with comprehensive and evidence-based information on dietary supplements, herbal-and drug-induced liver injuries, edited by the Liver Disease Research Branch of the National Institute of Diabetes and Digestive and Kidney Diseases in partnership with the National Library of Medicine [26].

– Low-phospholipid-associated cholelithiasis (LPAC): gallstone disease is linked to mutations in *the ABCB4* gene in young people (younger than 40 years old) with symptomatic intrahepatic biliary lithiasis before and later even after cholecystectomy. A worsened secretion of phospholipids in the bile decreases the solubility of cholesterol, promoting gallstone formation. For this reason, long-term therapy with UDCA prevents several complications, especially after cholecystectomy, such as secondary sclerosing cholangitis [27].

Single or compound heterozygous mutations, especially in the *ABCB4* gene, cause some diseases, including LPAC, ICP, BRIC, and DIC. The possibility of developing the disease clinically depends on the interaction with environmental factors and the presence of mutations in other cholestasis-associated genes.

## 4. Progressive Familial Intrahepatic Cholestasis-Related Genes

### 4.1. Role of Next-Generation Sequencing

Traditional molecular testing methods greatly relied on Sanger sequencing technology [28]. So far, this technique is still the gold standard for molecular diagnosis when the genomic region of the disease is known and it is made of a few coding exons. It is also used for variants’ validation from high-throughput sequencing.

In the last years, NGS technologies have led to advances in understanding genome architecture [29]. NGS has dramatically facilitated the integration of NGS-based genetic analysis strategies in clinical diagnostics processes. In addition, it offers good data quality and affordable costs, improving data handling capabilities and increasing the computational power and efficiency of bioinformatics analysis tools. NGS can be used broadly for variants’ discovery. Panel gene sequencing (PGS) and WES/WGS are the main applications in molecular diagnosis.

PGS involves the selective enrichment of genes or genomic regions, already suggested by other genetic analyses and already known to be associated with clinical disorders or biological pathways. If disease genes are known and their number is limited (<100), it is recommended to set a target analysis with PGS. WES, instead, covers approximately the 20,000 known protein-coding genes in the human genome [28].

Mendelian and complex diseases are mainly caused (over 85%) by defects in protein sequences [30]. When classical target genes do not reveal any variation in their coding sequences, a WES approach is recommended since variations in coding sequences of other genes are probably present. Compared to other traditional molecular clinical diagnostic tests, WES presents higher rates of molecular diagnostic success when used for common disease traits, non-specific phenotypes, and rare variants [31,32,33].

WES technology is useful in the identification of rare and de novo mutations in the analysis of trios or families [34]. For instance, the other cholestasis-related genes have been identified by this approach.

The recent spread of “omics” studies, the complete sequencing of the human genome, and the decrease in the cost of this technology (less than USD 1000 per genome in the Illumina NovaSeq or BGI/MGI platforms) have favoured the use of WGS in research and clinical genetic diagnosis [29,35]. The T2T-CHM13 assembly added five complete chromosomal arms and more additional sequences than any genomic reference released in the last 20 years. While 8% of the human genome was omitted in the past due to technological limitations, now high-precision long-read sequencing has finally removed this technical barrier. This will make it possible in the future to rapidly discover new genes and new genetic pathways underlying disease and health [30].

Most Mendelian diseases are due to deleterious mutations within the exome. However, it is possible genetic variations having significant clinical implications can occur outside the exome sequences, risking not being identified [36,37]. Using WGS, it is also possible to find variants caused by copy number variants (CNVs), short tandem repeats (STRs), DNA deletions and insertions (INDELs) and structural variants (SVs).

### 4.2. Hepatobiliary Cancers and Cholestasis-Related Genes: An Underestimated Association

Hepatocytes are cells involved in many metabolic cell functions. They are, therefore, subject to many injuries potentially resulting in abnormal proliferation leading to liver cirrhosis as well as HCC.

Chronic hepatitis B and C are the most common etiologies associated with HCC in adults, followed by alcoholic and nonalcoholic steatohepatitis [38].

On the opposite to what is observed in adults, in children, HCC often develops in a non-cirrhotic setting. In Western countries, pediatric liver cancers were reported to occur in patients with PFIC; notably, 5–15% of children with PFIC2 present HCC early, from 13 to 28 months of age [38,39,40], but several cases are well described in infants with *TJP2* disease (PFIC4) and MDR3 deficiency (PFIC3) [41,42,43,44].

In some instances, liver cancer is diagnosed incidentally during routine image exams, in young and adult people with unknown chronic liver disease, often because of mutations in critical genes involved in the metabolic pathways of hepatocytes or the metabolism and transport of BAs. As a result of *ABCB4* variants or other mutations in cholestasis-related genes, these subjects often present serum liver enzyme abnormalities, particularly alanine aminotransferase, aspartate amino-transferase and GGT, in the absence of a clear aetiology of liver injury [1]. About 20% of cases of HCC can develop in a non-cirrhotic liver presenting with two peaks during the 2nd and 7th decade of life [9]. Unfortunately, in these patients, data, especially about genetic risk factors, are missing. A late-onset disease, a slower progression of a liver disorder or a predisposition to further hepatic injury are all possible phenotypic manifestations when genes involved in inherited cholestatic diseases are affected with a single variant (heterozygotic state) or homozygous variants with a milder reduction in protein function, or a combination of both [8]. Therefore, the attention is recently focusing on variants of these genes associated with childhood diseases among adults with different pictures of cryptogenic cholestasis [1].

Large-scale whole-genome sequencing of the Icelandic population showed a strong association between some mutations in the *ABCB4* gene and the increased risk of liver diseases. In addition to gallstone disease, ICP, liver cirrhosis and increased serum liver-related exams (alanine transaminase, aspartate transaminase and GGT), the *ABCB4* variants were also linked to liver, gallbladder and gall ways cancer. A total of 681 cases were diagnosed with HBCs in Iceland from 1955 to 2011, identified by the nationwide Icelandic Cancer Registry. Four variants in *ABCB4* (c.1865G>A, p.G622E; c.1333_1334delCT, p.L445Gfs*22; c.1529A>G, p.N510S; c.711A>T, p.I237=) were associated with these liver-specific tumours [45].

Variants of *ABCB4*, together with those of the *PNPLA3* gene for fatty liver disease, are considered the most relevant common genetic determinants of the cholestatic response of the liver to injury. Therefore, given the increased risk of developing HBCs, especially young subjects with known mutations in the *ABCB4* gene, should undergo surveillance programs [46].

Interestingly, gene rearrangements in *ABCB11* deficiency, using *Mdr2*-KO mice, frequently target the mitogen-activated protein kinase signalling pathway and stress the mechanisms leading from the gene defects to liver cancers secondary to phosphatidylcholine deficiency and low-phospholipid concentrations in bile. They play as inducers of liver injury in the general population and not only in patients with rare liver diseases such as PFIC, ICP or LPAC [47].

Moreover, polymorphisms such as rs2109505 (c.711A>T, p.I237=) in *ABCB4* and rs2287622 (c.1331T>C, p.V444A) in *ABCB11* are more prevalent in adult patients with idiopathic cholestasis than in healthy controls representing risk factors for the development of liver fibrosis [48]. In addition, the variant p.V444A in *ABCB11* was observed more frequently in patients with DIC and ICP because of reduced liver BSEP expression [8].

In keeping with these findings, it could be speculated that many of the mild phenotypes concerning idiopathic cholestasis in adult people could be due to single or compound mutations in PFIC-related genes.

Pathogenic or likely pathogenic mutations in *ATP8B1*, *ABCB11*, *ABCB4* and *TJP2* genes can be responsible for 21% of idiopathic cholestasis in young and adult patients: they have higher rates of cholestatic histological features, higher levels of liver fibrosis and serum BAs compared to the subjects without, at least likely, pathogenic mutations [1].

The extensive development of diagnostic methods such as NGS and WES is leading to the discovery of numerous additional genes responsible for inherited cholestatic diseases and the application of historical gene panels related to child pathologies in the adult population with forms of cryptogenic liver disease.

It is unclear whether the prevalence of variants in cholestatic-related genes is as low in adults as in children.

Since some PFIC gene variants in *ABCB11*, *ABCB4*, and *TJP2* are described in patients with liver tumours, subjects with idiopathic chronic cholestasis and personal or familial risk factors for inherited cholestasis as well as DIC, ICP or LPAC history, should be screened for a panel of primary cholestasis-related genes and might likely benefit from monitoring with periodic ultrasound exams.

### 4.3. Hepatobiliary and Nor Cancers in Patients with Mutations in Cholestasis-Related Genes: PFIC Genes History and Cholestasis-Cancer Links

#### 4.3.1. ATP8B1

Clayton et al. first described Byler disease, a progressive form of intrahepatic cholestasis in Amish children leading to death in the first decade of life [49]. Patients presented early onset of loose and foul-smelling stools, short stature, jaundice, and hepato-splenomegaly. Since the bile showed an increased proportion of dihydroxy BAs, the changes in stool abnormalities occurred early in life and cholestyramine produced clinical improvement, a defect in bile salt metabolism was hypothesized. The children’s mother suffered from severe itching during the third trimester in all of the four pregnancies: this condition was then described as ICP.

In another child with Byler disease described by De Vos et al. (1975), liver histology showed intrahepatic cholestasis while electron microscopy highlighted interruptions of the canalicular bile membrane [50], suggesting a primary disturbance in BAs secretion as the cause of the cholestatic disorder.

Nielsen et al. (1986) described Byler disease in 16 Greenland Eskimo children [51]. Itching, jaundice, sarcopenia, steatorrhea, osteodystrophy, short stature and jaundice were frequent in children. Eight patients died before they were three years old while inheritance resulted in autosomal recessive.

Jacquemin et al. (1994) found a lower concentration of total BAs in the bile of 7 children with Byler disease while total BAs levels in serum were increased, so they concluded that a defect in primary BAs secretion was present in the disease [52].

In 1995 Carlton et al. mapped the disease locus to chromosome 18q21–q22 [53] (now become 18q21.31) in the Amish population. The discovery of this region was found by comparing the DNA of two distantly related Old Order Amish PFIC patients, and finding shared segments. In addition, they noted that a locus for BRIC had been mapped to the same region and suggested therefore that BRIC and PFIC were allelic disorders. It was 1998 when Bull et al. finally first described positional cloning in the *ATP8B1* (PFIC1) gene [54].

To the best of our knowledge, there are no cases of HBCs in patients with *ATP8B1* mutations.

#### 4.3.2. ABCB11

In 1976 Sandor et al. [55] first described two siblings (brother and sister) with ‘giant cell hepatitis’ in infancy. The man died of liver cancer with decompensated cirrhosis.

Strautnieks et al. (1998) [56] found patients with normal serum cholesterol and GGT levels with defective BAs transport at the hepatocyte canalicular membrane, identifying ten different mutations in the *ABCB11* gene encoding BSEP.

Knisely et al. (2006) [39] studied 11 unrelated children with PFIC and HCC diagnosed between 13 and 52 months of life. BSEP deficiency resulted immunohistochemically in 10 children, while materials from the children or their family members allowed the molecular diagnosis of mutations in *ABCB11*. The first case of HCC was found in the explanted liver of a boy with BSEP deficiency (in Table 2, we reported the cases described in PubMed of HBCs associated with mutations in cholestasis-related genes) who manifested at three weeks of age failure to thrive and jaundice.

Laboratory exams revealed hypercholanemia, conjugated hyperbilirubinemia, and normal GGT. Liver histology showed the absence of BSEP and identified neonatal hepatitis with intralobular cholestasis and anisocytosis, oedema, rosetting, multinucleation and cell necrosis of hepatocytes. After LT, the explanted liver revealed the presence of an HCC of 0.5 cm. Furthermore, mutational analysis of *ABCB11* highlighted compound heterozygosity for c.2012-8T>G (IVS16-8T>G), and c.1939delA (p.G648Vfs*6). Both mutations produce shorter and truncated proteins, respectively. 

Based on this experience, ten additional young patients with HCC associated with BSEP deficiency were identified at King’s College Hospital and elsewhere after reviewing cases of HCC in children with PFIC. All the subjects were unrelated (Table 2).

They had conjugated hyperbilirubinemia without biliary blockage. Moreover, the histology of 9 patients showed “neonatal hepatitis”. GGT levels and Bas synthesis disorder on urine screening were normal. Instead, serum alpha-fetoprotein (AFP) concentrations were elevated in eight subjects. Unfortunately, five of these patients died from HCC; the other 6 underwent LT. One died of sepsis shortly after LT. No HCC recurrence was observed in the other patients.

Genomic DNA for mutational analysis of *ABCB11* was obtained from the patient’s and parent’s peripheral blood. Table 2 reported clinical outcomes, nucleotide changes, and predicted consequences of mutations in the eleven children. No variant in *ABCB11* was associated in the study with the progression of HCC. Among the various expected effects (Table 2), the authors report missense, splicing, frameshift and stop codon mutations. Two missense variants (c.890A>G, p.E297G, c.1445A>G, p.D482G) have been reported previously [54] causing an alteration of BSEP transport activity [55,56]. Patient B was affected by IVS18 + 1G>A (c.2178 + 1G>A), previously reported in BRIC (in combination with c.3148C>T, p.R1050C) [57]. The variant IVS16-8T>G (c.2012-8T>G), found in patient A, leads to skipping exon 17 with an associated frameshift and introducing 5 amino acid residues followed by protein truncation. This mutation has been described in several other patients with PFIC. IVS13del-13ˆ-8 modifies the 3’ splice site, while IVS18 + 1G>A (c.2178 + 1G>A) and the other three splice site changes affect the GT 5’ donor splice. Patient B, with genotype IVS18 + 1G>A/c.74C>A (p.S25*), was affected by a PFIC phenotype while IVS18 + 1G>A/c.3148C>T (p.R1050C) genotype was previously reported in a less aggressive phenotype of a patient with BRIC [58]. Finally, the authors highlighted the presence among this cohort of the c.890A>G (p.E297G) mutation (patient D, homozygous; patients E and H, heterozygous) and the c.1445A>G (p.D482G) mutation (patient C, heterozygous), previous described in PFIC or BRIC subjects [59,60].

Scheimann et al. in 2007 [61] described two children with peripheral CCA, affected by BSEP deficiency. The *ABCB11* mutation c.1723C>T, p.R575*, previously described by Strautnieks et al. (1998) [56], was identified in a homozygous state in patient A and in a heterozygous state in her mother. In addition, the child and her father were carriers of a deletion of at least 12.5 Mb in one copy of chromosome 2. A total absence of functional BSEP was observed in patient A. Since peripheral-blood DNA from patient B was not available, compound heterozygosity in *ABCB11* was found in her mother, who presented low GGT intrahepatic cholestasis without HBCs: two mutations were c.890A>G (p.Q297G), previously reported by Strautnieks et al. (1998) [56] and c.2343 + 1G>T, a novel splice site change. Accordingly, BSEP was absent at immunohistochemical analysis performed in patient B and in her mother. Davit-Spraul et al. in 2010 [40] reported 5/36 PFIC2 children (26%) that progressed early to liver failure or HCC. Decompensated liver cirrhosis occurred before one year of age in three children, while HCC was observed as early as seven months of age in one patient.

Forty-one mutations in *ABCB11* were discovered: one novel synonymous (p.R1001=), seven indels, six nonsense and three intronic mutations. The 24 other variants were missense substitutions, such as p.V444A which was present on 44% of PFIC alleles analyzed. Seven children were heterozygous carriers. PFIC2 phenotype was assessed by mutations led to a truncated protein or when a missense variant was associated with low biliary BAs concentrations or the absence of BSEP at immunostaining.

The p.R698H mutation was identified in three children with negative or focal negative BSEP canalicular staining [62]. It was previously reported as a possible rare SNP by Lang et al. in 2007 [63]. Immunostaining for BSEP and MDR3 was available in 24 patients: BSEP was negative in 22 and focally negative in two, while MDR3 was regularly expressed in all subjects. AlSalloom et al. in 2013 [64] reported an 11-month-old Saudi boy with a known PFIC2 (confirmed by immunohistochemistry) admitted for LT. At explant analysis, histopathological examination revealed HCC, but no genetic analysis was performed.

Vilarinho et al. in 2014 [65] described a patient with a diagnosis of 2.5 cm HCC at 17 months of age. He has suffered from itching since the age of eight months. WES analysis revealed bi-allelic missense mutations in *ABCB11*, resulting in highly conserved amino acid regions (Table 2). The authors concluded that the bi-allelic *ABCB11* variant had led to the development of HCC in this patient. Moreover, WES in the HCC tissue revealed somatic mutations *CTNNB1* p.S33P and *NFE2L2* p.D27_L30delID that could better explain the rapid development of liver cancer.

In 2008 Strautnieks et al. [66] analyzed 109 families worldwide for germline mutations in *ABCB11* and hepatic expression of *ABCB11* to search for a correlation of genotype with the occurrence of HCC or CCA. There were 82 mutations in *ABCB11* identified, while bi-allelic variants occurred in 93% of families. The majority (55%) of mutations were missense. The mutations predicted to truncate the BSEP protein (comprising 45% of all variants) were distributed throughout the protein, whereas most missense mutations were clustered in 2 highly conserved nucleotide-binding fold domains. Most of the patients (93%) had abnormal or absent BSEP at liver immunostaining. Patients who developed HCC or CCA (previously reported by Scheimann et al. 2007 [61]) were 15% of cases: 38% of children with two protein-truncating mutations showed the highest risk of malignancy compared to 10% of patients without truncating mutations. This study shows that reduced or absent immunohistochemical detection of BSEP correlates well with the presence of mutations in patients with BSEP deficiency, and those subjects with protein-truncating mutations are at the highest risk for HBCs.

According to previous studies, a retrospective multicentre cohort study including 264 patients with pathological *ABCB11* mutations confirmed the increased risk of HCC occurrence according to the genotype severity.

Patients were categorized as:

– BSEP1, if carrying at least one of the two common European mutations associated with residual BSEP function (c.1445A>G or c.890A>G);
– BSEP2, if carrying at least one missense mutation, different to (c.1445A>G or c.890A>G);
– BSEP3, if carrying mutations causing non-functional protein.

At 15 years of age, the observed incidence of HCC was 4% in BSEP1, 7% in BSEP2 and 34% in BSEP3, respectively (*p* = 0.001). HCC was observed in 6% (1 patient with homozygous p.E297G), while there were no cases in the group with p.D482G mutations and in the three patients with compound heterozygous p.D482G/p.E297G variants [67].

#### 4.3.3. ABCB4

MDR3 glycoprotein is a phosphatidylcholine flippase sited in the canalicular membrane of hepatocytes. MDR3 protects the cholangiocytes from the detergent activity of BAs, carrying phosphatidylcholine from the hepatocytes into the bile canaliculus.

In a study by Deleuze et al. (1996) [68], the authors found no MDR3 mRNA by Northern blotting in the liver of a patient suffering from a form of PFIC disease associated with increased serum levels of GGT while the biliary phospholipid levels were substantially low in a second patient. In addition, liver histology showed portal inflammation and ductular proliferation.

De Vree et al. (1998) described two unrelated children with ABCB4 disease [69]: a Turkish boy with consanguineous parents, who resulted affected from the age of 3 months when he presented diarrhoea, fever, and itching. At 3 years, he had hepatosplenomegaly, high serum transaminase, GGT and BAs concentration. A biopsy showed liver with portal inflammation and extensive portal fibrotic septa. He had no response to treatment with UDCA, so LT was performed at the age of 3.5 years. The second patient, a North African boy born to first-cousin parents, had recurrent itching from 8 months of age. He developed hepatosplenomegaly at three years of life and maintained high GGT and serum BAs (16 times higher than upper normal limits). Liver histology showed ductular proliferation and cirrhosis. LT was performed at the age of 9 years. Among family members, the child’s mother experienced recurrent ICP.

Regarding the association between the *ABCB4* gene and HBCs, Wendum et al. [70] first described adult PFIC3 patients developing liver carcinoma in 2012. They reported an analysis of thirteen patients (five men, eight women) who had a gene mutation in the *ABCB4* gene and a histopathological study. One female patient had mild periportal fibrosis and ductular reaction. Moderate biliary dysplasia was identified in large bile ducts. She developed an intrahepatic CCA (Table 2) one year after the first partial hepatectomy because of hepatolithiasis with several episodes of recurrent cholangitis. Another patient had liver failure secondary to biliary cirrhosis leading to LT. A 4-cm liver nodule was identified on the explanted liver with areas of well-differentiated HCC. Both patients had a heterozygous *ABCB4* mutation: c.1005 + 5G>A (splicing) and p.S320F (pathogenic missense), respectively (Table 2). Immunohistochemical staining of MDR3 showed a diffuse and strong canalicular pattern.

Poupon et al. 2013 [71] studied 156 consecutive patients with LPAC syndrome. After *ABCB4* sequencing, 79 patients of 156 subjects had mutations (61 missense and 18 truncating sequence variants). All the patients with a truncating variant had one allele mutated, while four cases were compound heterozygotes. In the overall cohort, only two patients had liver cirrhosis with c.523A>G (p.T175A) and c.959C>T (p.S320F) variants in both cases and only a non-cirrhotic female experienced intrahepatic CCA with a heterozygous splicing mutation (c.1005 + 5 G>A). Vij et al. 2015 [72] described the first case of pediatric HCC in a severe PFIC3 by histologic analyses without performing gene sequencing.

More the 200 of 8258 Icelandic patients with gallstone disease enrolling in a study using GWAS to search for predisposing causes were diagnosed before 40 years [45]. The GWAS revealed an association of missense and splice region variants in *ABCB4* with gallstones. In addition, the *ABCB4* mutations were also associated with cholestasis in pregnancy, liver, gallbladder and bile duct cancer, cirrhosis and increased serum levels of liver-related biomarkers, including transaminases and GGT. Among 303 cases of cirrhosis and 681 cancer cases four *ABCB4* variants were identified (c.1865G>A, p.G622E; c.1333_1334delCT, p.L445Gfs*22; c.1529A>G, p.N510S; c.711A>T, p.I237=) associated with these liver-specific traits (Table 2).

However, when *ABCB4* gene variants were analyzed in adult carriers, there was a limited risk of CCA. Two patients died of CCA, while three additional deaths of CCA were reported in first-degree relatives, one of them having a proven *ABCB4* variant in a cohort of sixty-seven patients. Transplant-free survival of the study population was 91% (median follow-up of 14 years) and liver stiffness was normal (<6.3 kPa) in 75%. The two patients presented LPAC syndrome, and they died at the age of 71 years with intrahepatic CCA and at the age of 47 with perihilar CCA, respectively. The mutation involved were c.1405A>T, p.R469W and c.1268A>C, p.Q423P (Table 2) [73]. In the same study cohort, one patient with LPAC syndrome needed an LT because of decompensated cirrhosis, receiving a diagnosis of HCC in the explanted liver (mutations c.760G>A, p.A254T; c.1546A>G, p.M516V; c.2363G>A, p.R788Q; Table 2). The authors hypothesized a potential association between the chronic inflammation in the biliary tree and the occurrence of dysplasia-carcinoma such as in other models of cholestatic disorders including the primary sclerosing cholangitis [73].

#### 4.3.4. TJP2

Sambrotta et al. (2014) identified homozygous mutations of *ABCB4* in 12 cholestatic children from 8 families by combining WES and targeted sequencing of cholestasis-candidate genes [43]. GGT were normal or mildly increased and most of the families were consanguineous. Phenotypes of patients were heterogeneous:

– Nine patients required LT;
– One child died at 13 months;
– Two had stable liver disease with mild portal hypertension at the ages of 4 and 7 years.

The first association between *TJP2* and HCC was revealed by Zhou et al. (2015) in two patients [42]. One of the two children was a 26-month-old Caucasian female with neonatal cholestasis of unknown aetiology and normal GGT. Computed tomography revealed multiple liver nodules in a patient with impressively high levels of AFP (171,000 ng/mL): liver biopsy found moderately differentiated HCC in a background of chronic cholestatic cirrhosis. Both BSEP and MDR3 were well expressed, while the authors did not find significant variants in *ABCB11*. *TJP2* sequencing revealed compound heterozygous mutations c.2668-1G>T/c.2438dupT (Table 2).

The second case was a 6-month-old Caucasian male referred for persistent cholestasis with normal GGT after Kasai surgery for presumed biliary atresia. Jaundice resolved by age 19 months, but LT was performed at age of 2 years for the occurrence of a single HCC 2 cm tumour secerning AFP. Since no mutations were found in *ABCB11*, WES was performed on patients’ blood and tumour revealing homozygosity for c.817delG (p.A273Pfs*38) in *TJP2.* This mutation was predicted to cause a frameshift in all protein transcripts (Table 2) while expressions of TJP2 and claudin-1 were respectively absent and markedly reduced in nontumoral liver tissue

Another reported case is a 7-year-old girl with a history of jaundice and itching who received the diagnosis of PFIC at the age of one year after a liver biopsy [74]. She underwent partial internal biliary diversion in 2012 at the age of 3 years. Unfortunately, her liver disease worsened over the next few years, so a split LT was performed when her paediatric end-stage liver disease (PELD) score was 31. Histologic exam of the explanted liver showed an early well-differentiated HCC measuring 1.2 cm in addiction to regenerative nodules. NGS allowed the discovery of the homozygous deletion of exon 18 of the *TJP2* gene, from an unknown position in intron 17 (c.2659 + 1_2660-1) to an unknown location in intron 18 (2760 + 1_2761-1). No mutations were identified in *ABCB11*, *ABCB4* or *ATP8B1* genes.

Instead, only a case of *TJP2*-related HCC was reported in the adult population: a 19-year-old affected by novel homozygous mutation (c.3334C>T, p.Q1112*) developed cirrhosis, variceal bleeding and primary liver cancer: despite receiving variceal endoscopic band ligation and beta-blockers as secondary prophylaxis of variceal bleeding and radiofrequency ablation for the liver tumour, he needed LT later on (Table 2) [75].

#### 4.3.5. NR1H4

*NR1H4* was discovered by Forman et al. in 1995 [76]. In rat liver, it encodes for two isoforms of FXR (FXRα and FXRβ), FXRα, the primary BAs receptor, is the only isoform expressed in humans, while FXRβ is a non-expressed pseudogene [77]. FXR is mainly located in the liver and intestine, to keep the homeostasis of BAs throughout the regulation of the uptake, synthesis, conjugation and transport. In 4 infants from 2 unrelated families with PFIC5, Gomez-Ospina et al. (2016) discovered homozygous or compound heterozygous loss of function mutations in the *NR1H4* gene [78]. The mutations found by WES and SNP array analysis segregated with the disorder in the families. The mutation p.R176* had previously been identified in the heterozygous state in a Chinese infant with cholestasis and increased GGT [79]. However, the heterozygous parents in the first family reported by Gomez-Ospina et al. (2016) had normal liver blood exams, and the mother did not have symptoms of cholestasis during any of her three pregnancies, suggesting heterozygosity for p.R176* is not sufficient to cause PFIC and that severe condition may be due to other untested genes [78].

FXR downregulates the expression of the rate-limiting enzymes cholesterol 7α-monooxygenase (CYP7A1). Other steps of BAs metabolism and transport regulated by FXR are:

– The increase in the synthesis of fibroblast growth factor-19 (FGF-19);
– The CY7A1 inhibition through the fibroblast growth factor receptor 4 (FGFR4) pathway in the hepatocytes (Figure 1);
– The downregulation of sodium taurocholate cotransporting polypeptide (NTCP) blocking the uptake of BAs by the liver;
– The upregulation of the synthesis of BSEP and MDR3;
– The increase of BAs efflux from the liver to the lumen of bile canaliculus;
– The synthesis of organic solute transporter alpha/beta (OSTα/β) via enhancement of BAs output from the liver to the portal vein [80].

The absence of reports of HBCs in patients affected by mutation in the NR1H4 gene may be explained because the few cases described to date present a neonatal onset and a rapid evolution to liver failure. For example, in the first report, two patients died at 8 months and 5 weeks, while LT was performed at 22 months and 4.4 months in the other two cases, respectively [78]. In another article, two children died at 6 and 8 months of life, while a male infant received LT at 20 months [81]. The potential key role of the FXR in HBCs occurrence can be explained by the BAs synthesis inhibition reported in the *Fxr*^−/−^ mice, which present increased BAs and proinflammatory cytokines, failure proceed to apoptosis and cell hyperproliferation, resulting in the development of liver cancers [82]. Contrarily, persistent stimulation of FXR in *Abcb4*^−/−^ mice by administration of the FGF19 analogue INT-767, derivate from obethicolic acid, can reduce BAs levels, re-program the BAs metabolism, avoid progression of liver fibrosis, cellular proliferation and the occurrence of HBCs [83,84].

Consistently with these results, when FXR expression was tested in intrahepatic CCA and HCC tissues and cell lines, reduced protein levels were linked to cell proliferation, migration and invasion, which are all markers of poorer prognosis [85,86].

Interestingly, increased intestinal BAs are a risk factor for colorectal cancer; strong evidence suggests a role for FXR in gut tumorigenesis, with expression levels inversely related to colon-rectal cancer progression and malignancy. Conversely, the activation of FXR in the intestine reduces cancer severity and increases survival [87].

These findings warrant research on the therapeutic use of the FXR agonists in preventing HBCs and intestinal cancers in patients with inherited and acquired cholestasis.

#### 4.3.6. MYO5B

Until a few years ago, *MYO5B* was associated only with MVID. However, 8 among 28 MVID patients in the Girard et al. (2014) cohort developed a cholestatic liver disease up to PFIC in some instances [88]. Patients were affected by cholestasis before (n 5) or after (n 3) gut transplantation and they had intermittent jaundice, intractable itching, elevated serum BAs, and regular GGT activity. Histology showed canalicular cholestasis, different stages of fibrosis and ultrastructural alterations of bile canaliculi. Portal fibrosis was present in 5 patients.

Gonzales et al. (2017), using an NGS approach, identified *MYO5B* mutations in five patients with PFIC-like phenotype and normal serum GGT without intestinal disease [89]. They showed for the first time that *MYO5B* deficiency may lead to isolated cholestasis and that the MYO5B gene could be considered an additional PFIC locus.

*MYO5B* downregulation, mutations and epigenetic changes have been associated with colon-rectal, bladder and gastric but not liver cancers. One of the first discoveries was made by Kuang et al. in 2008. The authors found that DNA methylation and histone deacetylation of eight genes, including *MYO5B*, are associated with suppressed gene expressions in leukaemia cell lines. Furthermore, patients with methylation of multiple CpG islands had worse overall survival [90].

Dong et al. 2012 [91] analyzed MYO5B, Rab11a and TfR expression in 70 human gastric tumour tissues by immunohistochemistry using a tissue microarray method. They found that MYO5B was repressed in 78.6% and 17.1% of gastric cancer and normal gastric tissues (*p* < 0.001). In addition, the MYO5B expression correlated strongly with the Rab11a expression (*p* = 0.002). They also showed that *MYO5B* inactivation by siRNA facilitated the proliferation, invasion and migration of gastric cancer cells.

#### 4.3.7. SLC51A

Solute carrier family 51 alpha subunit (*SLC51A*) encoding the OSTα-OSTβ, an essential contributor to intestinal BAs reabsorption in the enterohepatic circulation. Gao et al. (2020) reported a case of a child born to consanguineous Pakistani parents with chronic diarrhoea, bowel malabsorption, easy bruising, persistent bleeding and failure to thrive. Laboratory exams at age 2.5 years showed increased transaminases and alkaline phosphatase. Liver histology documented portal and periportal fibrosis and hepatocytes foci of hepatocytic cholestasis. BAs levels were normal. By WES, confirmed by Sanger sequencing, they identified a homozygous mutation in the *SLC51A* gene (p.Q186*) [15]. The variant segregated with the disorder in the family. Analysis of the patient’s colon tissue showed an absence of SLC51A expression. After this study, OMIM coded for another cholestasis phenotype, named PFIC6.

#### 4.3.8. USP53

In 2019 Maddirevula et al. reported the first three cases of PFIC7 due to mutations in the *USP53* gene in a consanguineous Saudi family with low GGT pediatric-onset cholestasis. One child underwent LT at the age of six years. However, three patients, a child and two adults had not a progressive liver disease but recurrent episodes of normal GGT-cholestasis and BRIC phenotype. In addition, the authors remarked on an excellent response to rifampicin.

In 2020, Zhang et al. described seven unrelated Chinese children with jaundice until seven months; one patient also had hearing disorders, and no patient needed LT.

Lastly, Bull et al. reported in 2021 4 unrelated patients with a variable course of cholestasis without deaths or LT occurrence. Changes in *USP53* cause instability of tight junctions in the hepatocytes [17,22,92]. *USP53* gene has been associated with some models of tumorigenesis:

– In clear cell renal cell carcinoma inhibits the occurrence and development of cancer through NF-κB pathway inactivation [93];
– In cervical squamous cell carcinoma correlated with the sensitivity to radiotherapy [94];
– In oesophagal carcinoma, *USP53* suppresses cancer progression by regulating cell growth and metabolism [95];
– In lung adenocarcinoma, *USP53* regulates cell apoptosis and glycolysis through the AKT1 pathway acting as a tumour suppressor [96].

However, associations between *USP53* with HBCs have not yet been reported.

#### 4.3.9. KIF12

In 2019, Unlusoy Aksu et al. described three patients with PFIC features, high GGT and BAs, neonatal cholestasis and mutations in the *KIF12* gene [97]. In the same year, Maddirevula et al. reported four patients with a similar history of elevated GGT and neonatal cholestasis [17].

In 2022 Stalke et al. reported six patients aged 5 to 13 years from four unrelated families with high GGT. Two patients developed cirrhosis and received LT at age of 12 and 4 years. The other three patients developed portal hypertension or cirrhosis at the ages of 5, 12, and 13 years. PFIC8 follows autosomal recessive inheritance, and *KIF12* mutations cause cholestasis by changes in hepatocyte polarity [18].

This newly identified locus expands the genetic heterogeneity of pediatric cholestatic liver disorders and highlights the wide range of molecular perturbations in the bile homeostasis process. No HBCs are reported in the study population.

Finally, some phenotypes described by the authors could only partially fit into the classic definition of familial intrahepatic cholestasis.

#### 4.3.10. SLC25A13

Neonatal intrahepatic cholestasis caused by citrin deficiency (NICCD) is an autosomal recessive disorder. Pathogenic mutations of the *SLC25A13* gene, encoding the calcium-binding protein citrin, cause NICCD. Citrin is an aspartate–glutamate carrier located within the inner mitochondrial membrane having a key role in several metabolic pathways, including protein, nucleotide and urea synthesis (Figure 1) [98].

NICCD initially presents as neonatal intrahepatic cholestasis and often resolves within the first year of life. However, some children may have persistent high AFP levels, develop advanced liver disease and severe infections, or have the symptoms of adult-onset citrin deficiency [99]. Failure to thrive and dyslipidemia caused by citrin deficiency (FTTDCD) is a novel post-NICCD phenotype, but its clinical features are still being established. He et al. 2019 [100] described a 2-year-old girl with elevated serum levels of transaminases, alkaline phosphatase, GGT, total and direct bilirubin, BAs, and triglyceride. However, the serum level of high-density lipoprotein was decreased, indicating the existence of cholestasis and dyslipidemia. At the same time, AFP was constantly very high (25.283, 674 and 425 ng/mL, respectively, at 2, 4 and 5 years of age). Liver histology at the age of two showed cirrhosis with regenerative nodules, microvesicular steatosis, bile duct proliferation, and infiltration of inflammatory cells. In addition, the genetic test revealed homozygous known frameshift mutation c.852_855delTATG (p.M285Pfs*2) in *SLC25A13*. During the follow-up, a magnetic resonance imaging at five years of age showed multiple liver nodules with diffusion restriction on diffusion-weighted imaging, suggesting a multifocal HCC [101].

Fatty liver has been frequently found on liver histology in patients with citrin deficiency. The proliferation of bile ducts and infiltration of inflammatory cells in the portal tracts were also observed. In the scenario of *SLC25A13* disorders, an association in vitro and in vivo with HCC was established. A high incidence of HCC was found, especially in the Japanese population with adult-onset type II citrullinemia (8 cases of the 56 adult-onset cases reported, approximately 14%). Consistently with the latter, the addition of citrulline into primary cultured hepatocytes of rats increased the incidence of epithelial cell foci in cancer promotion [102].

To confirm the relationship between *SLC25A13* disease and liver cancer, the homozygous c.852_855del mutation was discovered in a Taiwanese man with HCC at 48 years. At liver histology, in addition to a grade 2 HCC, chronic persistent hepatitis with moderate steatosis was diagnosed (Table 2) [101].

In another case, a 50-year-old male patient affected by citrin deficiency exhibited liver cancer with dual HCC and CCA mixed phenotypes (Table 2) [103].

A child with FTTDCD and the same homozygous frameshift mutation in *SLC25A13* [100] who exhibited neonatal intrahepatic cholestasis and persistent high transaminases levels received the diagnosis of advanced HCC at the age of 6 years. At hospital admission, laboratory abnormalities were detected: while plasma ammonia levels were normal, GGT was 221 IU/L and AFP was 14323 ng/mL [104].

The results in these cases support that both citrin deficiency and steatohepatitis may contribute to hepatocarcinogenesis [102].

#### 4.3.11. JAG1 and NOTCH2

Mutations in *JAG1* and *NOTCH2* genes are responsible for Alagille syndrome (ALGS1 and ALGS2, respectively), an autosomal dominant disorder defined by a paucity of intrahepatic bile ducts, combined with cholestasis, cardiac disorders, skeletal abnormalities, ocular malformations and facial dysmorphisms. Cell–cell Jagged/Notch interactions are critical for the early phases of cell differentiation (Figure 1) [105].

The ligand–receptor link induces proteolytic cleavage of the Notch receptor and the Notch intracellular domain (NICD) release. The NICD migrates into the nucleus; here activates recombining binding protein suppressor of hairless (RBPJκ), thus upregulating the transcription of Notch target genes, including locus for hepatocyte nuclear factor-1beta (*HNF1B*).

JAG1 is the primary ligand able to activate the Notch way during liver development between 56 days and 14 weeks of gestational age. Notch-expressing hepatoblasts, located in the liver tissue interface of the nascent portal tract, get signals from neighbouring mesenchymal cells expressing *JAG1* and respond by over-expressing *HNF1B*.

Abnormalities in Notch signalling were previously described in melanoma, glioblastoma, pancreatic, breast, ovarian cancers and HCC [106]. Notch signalling is involved in liver progenitor cell differentiation in the setting of cirrhosis with increased inflammation and fibrosis.

Persistent upregulation of this repair pathway stimulates malignant mutation of hepatic progenitor cells into cancer stem cells [107,108,109].

In *JAG1* pathogenic variants typical of Alagille syndrome, the absence of functional protein creates hepatic progenitor cells with an intermediate hepatobiliary phenotype, unable to differentiate into biliary cells, resulting in ductopenia and cholestasis [109]. Finally, the persistent over-activation of Notch way in hepatic precursor cells leads to downstream RBPJk-dependent transcription activity, failing repair cell damage, induction of liver fibrosis and secondary HCC (Figure 1) [6].

HCC were reported in patients with features of Alagille syndrome (and in some cases even without liver involvement), suggesting a correlation with mutations in *JAG1*-*NOTCH2* genes and HBCs by interfering with Notch signalling [110,111,112,113,114,115].

A review of literature conducted by Schindler et al. identified 21 cases of HCC in children and 13 in adults with Alagille syndrome. A lack of intrahepatic bile ducts and cholestasis before developing HCC was invariably present in children and adults, except for 1 adult patient who did not present a liver involvement; in the pediatric population, 16 of 18 children had cirrhosis, while only 4 of 9 adult cases showed an advanced stage of fibrosis (Table 2). However, many of the cases of HCC described in Alagille syndrome date back to before the discovery of the *JAG1* gene in 1997, and genetic tests were available only in five patients [116].

In conclusion, these findings underline the need for genetic screening of family members and the regular surveillance in patients with Alagille syndrome associated with *JAG1*/*NOTCH2* variants, including subjects with or without mild liver involvement.

#### 4.3.12. HNF1B

The homeodomain-containing superfamily of transcription factors includes *HNF1B*, also known as transcription factor-2 (TCF2). Expression of HNF1B is observed in many tissues including the kidney, liver, bile ducts, genital tract, pancreas, lung and gut.

In kidney cells, *HNF1B* regulates renal tubulogenesis while a reduction of HNF1B in the liver induces glucose intolerance, impairs insulin sensitivity and causes gluconeogenesis. Conversely, liver overexpression improves insulin sensitivity [117].

Germline mutations of *HNF1B* predispose to renal cancers. *HNF1B* acts as a tumour suppressor gene in cell carcinogenesis via *PKHD1* inhibition, the gene responsible for autosomal recessive polycystic kidney disease. The inactivation of *HNF1B* results in cell proliferation, distortion of the orientation of tubule cells, tubule dilation and cyst formation [118].

In keeping with its functions, mutations in *HNF1B* cause maturity-onset diabetes of the young (MODY) and renal cysts and diabetes (RCAD) syndrome, where diabetes occurs with renal cysts and/or other urogenital disorders.

A paucity of interlobular bile ducts was reported in a few infants with cholestasis, high GGT values and *HNF1B* mutations similarly with Alagille syndrome [119].

Only a single case of HCC and *HNF1B* was described: an infant having renal disease and progressive cholestasis from early months of life, developed a large liver cancer at 16 months, associated with an elevated AFP level. He underwent LT successfully; the explanted liver was cirrhotic. After the exclusion of pathogenic variants in main inherited cholestasis-related genes, a hemizygous de novo deletion of *HNF1B* on chromosome 17 between base pairs 34,817,422 and 36,242,969 was found (Table 2) [120].

In a retrospective cohort of patients, including 183 HCC and 69 intrahepatic CCA, the overexpression of HNF1B by immunohistochemistry was an independent prognostic factor for overall survival and disease-free survival of HCC patients but not ICCAs patients.

High HNF1B expression in HCC with biliary phenotype showed a poorer prognosis, confirming the role of *HNF1B* in the hepatobiliary differentiation of hepatoblasts to cholangiocytes during the carcinogenesis process [121].

### 4.4. Other DNA Changes

Not only do mutations affect the oligonucleotide chain, but other functionally relevant genomic changes can also occur in eukaryotic cells. Examples of mechanisms that produce such changes are explained by epigenetics (e.g., DNA methylation and histone modification) and DNA damage, referring to physical or chemical changes to DNA in cells. Such changes can also affect genetic information. A variety of exogenous and endogenous insults can promote DNA damage. The most important agents are chemicals, radiations, reactive oxygen species (ROS) and topological changes. They cause different types of DNA injury [121]. Depending on the nature of the lesion, cells found specific pathways to repair and resolve the DNA damage. The DNA double-strand break (DSB), one of the most studied lesions, can be mutagenic since chromosomal rearrangements or loss or gain of genetic information can occur during erroneous DNA repair [122]. ROS are known to be DNA damage mediators. For example, ionizing radiation induces DSBs through several mechanisms. Two of the most known are the direct high-energy damage to the sugar backbone of DNA and the free radicals generation in cells. ROS have also been reported to directly cause other forms of DNA injury through oxidizing nucleoside bases, possibly forming 8-oxo guanine and 8-oxo-7,8-dihydroguanine [123,124] which can lead to G-T or G-A nucleotide transversions if unrepaired. The base excision repair (BER) pathway generally detects and repairs oxidized bases. Still, when they co-occur on opposing strands, this mechanism can lead to the generation of DSBs [125]. ROS storage in the cells also leads to lesions, strand breaks and degradation of mitochondrial DNA.

Regarding pyrimidine, Thymidine glycol (Tg) is another type of oxidatively induced lesion in DNA. Tg can result from the direct oxidation of thymidine in DNA. However, it is not easy to know the exact distribution of such sites in the human genome. Tang et al., have tried to map Tg in human cells very recently [126]. They presented a novel DNA−protein cross-linking sequencing (DPC-Seq) method which relies on the NTHL1-mediated removal of Tg and a multistep procedure for the highly selective and efficient enrichment of DNA-NTHL1 cross-link for subsequent library construction and NGS analysis. NTHL1 is a DNA N-glycosylase of the endonuclease III family working on DNA substrates containing oxidized pyrimidine residues. According to their results, Tg was depleted in genomic regions linked with active transcription but enriched at nucleosome-binding sites [126]. This area is poorly understood, necessitating increased efforts and all these mechanisms are likely underestimated in mendelian or multigene diseases such as PFIC.

## 5. Bile Acids and Liver Cancer: Pathophysiology

### 5.1. Structural and Functional Hepatocyte Polarity and Liver Disease

Bile canaliculi are separated from sinusoidal blood by a wall of hepatocytes. The polarity of hepatocytes is a pivotal feature in ensuring essential functions of liver cells, requiring synergy between cell adhesion molecules, cell junctions, cytoskeleton, intracellular signalling strictures and extracellular matrix. Anomalies of tight junctions and biliary transporters are involved in PFIC diseases, including canalicular ATP binding cassette (ABC) pumps. They all finally lead to hepatocyte depolarization.

At the same time, proteins involved in intracellular protein trafficking, such as Rab11a, recycling endosomes in the Golgi Network, have a crucial role in determining and maintaining the correct polarity of hepatocytes [122].

In the large spectrum of inherited cholestatic disorders, documented protein defects involved in the inherited disorders of cell polarity are:

– Claudin 1 (NISCH syndrome) and *TJP2* (involved in PFIC4 and familial hypercholanaemia) in the group of tight junction proteins;
– *VPS33B* (ARC syndrome) and Myosin 5B (involved in MVID and PFIC) in the intracellular trafficking protein;
– *ATP8B1* (PFIC1), *ABCB11* (PFIC2), *ABCB4* (PFIC3), and *ABCC2* (involved in Dubin Johnson syndrome) in the canalicular membrane transporters;
– *SLCO1B1* and *SLCO1B3* among the basolateral membrane transporters (Rotor syndrome), localized to the sinusoidal membrane of hepatocytes and able to control sodium-independent cellular uptake of bilirubin glucuronide, BAs, steroids and drugs;
– *USP53* colocalizes with the tight junction proteins TJP1 and TJP2 in polarized epithelial cells, necessary to guarantee the stability of auditory hair and liver cells [122,123];
– *KIF12* is able to impair the hepatocyte polarity by interacting with MRP2 protein (*ABCC2* gene), which has a strong cytoplasmic signal, in PFIC8-affected liver [17].

Beta-catenin, a structural part of the protein of adherence junctions, is needed to maintain the epithelial polarity. Beta-catenin transmits the contact inhibition signal to the nucleus. Mutations in *CTNNB1* lead to intracytoplasmatic and nuclear abnormal storage of beta-catenin, following the upregulation of the Wnt signalling pathway. *CTNNB1* gene mutations were identified in children with hepatoblastoma and patients with HCC in up to 20% of cases, including a patient with PFIC2 [65].

However, *CTNNB1* mutation alone does not seem to induce liver carcinogenesis since only about 50% of hepatocellular adenomas containing beta-catenin activating variants evolve into liver cancer over time, suggesting the presence of other oxidative stress response pathways necessary to promote carcinogenesis in the liver in collaboration with beta-catenin/Wnt signalling [124].

In summary, polarity defects are present in familial intrahepatic cholestasis, as well as in HCC, where the polarity appears involved in the cancer pathogenesis [122].

### 5.2. The Gut Microbiome–Bile Acid Axis in Hepatocarcinogenesis

Recent reports showed the close relationship between the bacterial microbiota, the regulation of BAs metabolism and the development of HBCs.

In PFIC diseases, where the disorders of BAs metabolism and transport are genetically present, microbiota balance has a key role in the promotion of liver and biliary cancers given the anatomical connection between the liver and the gut via the portal vein [2].

Microbiota is involved in transforming primary BAs into secondary and tertiary metabolites. Deconjugation, dehydroxylation, oxidation and desulfation of BAs are the main mechanisms through which BAs modulate cell metabolism and inflammatory response. The two primary BAs-sensing receptors, FXR and TGR5, have been claimed to be involved in the development of HBCs. High levels of BAs induce tumour genesis via hepatic inflammation, oxidative stress, liver fibrosis, impaired mitochondrial function, DNA damage, resistance to apoptosis and cell hyperproliferation [2].

Abnormal expression of FXR alters the balance of BAs homeostasis, leading to hepatocarcinogenesis; FXR expression is decreased in human HCC samples and *Fxr*^−/−^ mice can spontaneously develop HCC through the activation of the Wnt/β-catenin signalling pathway [125,126].

Instead, TGR5, located in liver sinusoidal cells, gallbladder cells and Kupffer cells, is a negative regulator of the HCC envelope through its anti-inflammatory properties and ability to guarantee correct homeostasis of BAs [2].

The synthesis of BAs is a complex process starting from cholesterol and involving 17 different liver enzymes. The primary BAs, cholic acid and chenodeoxycholic acid, are conjugated with glycine or taurine in the liver and then reversed to their original forms in the small intestine before returning to the liver in 95% of the total amount in the so-called “enterohepatic circulation”. During this process, the primary BAs are transformed into secondary BAs by gut bacteria. The deconjugation of BAs via microbiota activates FXR and TGR5 receptors, whose correct functioning has a crucial role in the reduction of mucosal injury, ileal barrier permeability, bacterial overgrowth and translocation in animal models [127,128].

Interestingly, the butyrate-producing bacteria (*Ruminococcus*, Oscillibacter, Faecalibacterium, Clostridium IV and Coprococcus) decreased in patients with early HCC in comparison to healthy controls, whereas genera producing lipopolysaccharides (*Klebsiella* and *Haemophilus*) resulted increased [129].

Butyrate has a protective action against gut permeability, while lipopolysaccharides are involved in inducing some inflammatory responses to dysbiosis.

Dysbiosis can interfere with the metabolism of BAs, increasing their levels in systemic and portal circles, promoting the release of senescence-associated substances in hepatic stellate cells, the activation of FXR signalling, the stop of CYP7A1 expression and the small heterodimer partner (SHP) pathway, and the upregulation of angiopoietin-like 4. Changes in these pathways cause the increase in fat production, associated with steatohepatitis-related HCC [130,131,132].

Furthermore, butyrate influences the immunological response of the liver against cancer proliferation; the inhibition of FXR, by reversion of conjugated BAs via butyrate, controls the accumulation of hepatic natural killer T (NKT) cells and favours an NKT-related antitumor effect [133]. Some bacteria, such as *Erysipelotrichaceae* can produce butyrate that reverses the synthesis of conjugated BAs deoxycholic acid and β-muricholic acid in hepatitis and in liver cancer [134].

However, the data on butyrate in liver carcinogenesis are controversial. In fact, prolonged exposure to butyrate should promote hepatocyte proliferation and liver fibrosis [135]. However, high bilirubin values, hepatic inflammation, upregulation of liver fibrosis and HCC markers were found in a large group of butyrate-treated mice, but no tumours were observed. In the same study, HCC was found in dysbiotic mice but not in germ-free or antibiotic-treated mice. At the same time, dysbiosis, cholestasis and liver cancer were associated with an inulin-enriched high-fat diet in *Tlr5*-deficient mice. On the other hand, the blocking of enterohepatic circulation of BAs with cholestyramine and the removal of fermenting bacteria able to produce short-chain fatty acids starting from dietary soluble fibres prevented the onset of liver tumours. Surprisingly, cohousing of the dysbiotic *Tlr5*-deficient mice with wild type mice demonstrated the transmission of certain oncogenic bacteria including *Clostridium* cluster XIVa, transmitted to susceptible mice. Such findings point to the relationship between the gut microbiome, liver cancer and diet [135].

Accordingly, the use of antibiotics, such as oral vancomycin, suppresses the senescence-associated secretory phenotype of hepatic stellate cells and reduces the production of liver cancer-promoting factors including interleukin-6, growth-regulated oncogene-alpha and prostaglandin E2 by reducing the account of the gut microbiome [4,136].

In a mice model of steatohepatitis induced by a high-fat diet, treatment with antibiotics significantly reduced cancer development, indicating the central role of the gut microbiota in tumour carcinogenesis. BAs were increased in mice livers after feeding them with a high-fat diet. In contrast, secondary BAs were dramatically reduced after antibiotics treatment through the block of conversion of primary to secondary BAs produced by the gut microbiota.

In this model, secondary BAs activated the mammalian target of the rapamycin (mTOR) pathway, linked to liver carcinogenesis [3]. Conversely, hydroxylation of BAs is a common mechanism for their detoxifying effect. Therefore, hydrophilic BAs such as tetrahydroxylated bile acids (THBAs) are more hepatoprotective and less toxic when compared with the usual BAs, which are typically di- or tri-hydroxylated. THBAs can inhibit BAs-induced liver injury in mice models [137].

In an activated Mdr2 (an ortholog of human *ABCB4* gene) mouse model for PFIC3, hydrophobic BAs toxicity induces liver injury such as in PFIC3 patients with cholangitis, ductular proliferation, periportal fibrosis, susceptibility to the formation of gallstones and HBCs occurrence. Instead, *Bsep*^−/−^ mouses generated high levels of hydrophilic BAs. High levels of THBAs and low levels of hydrophobic cholic acid were found in double-KO (*Bsep*^−/−^ and *Mdr2*^−/−^) mice. THBAs partially alleviated damage in their livers, preventing the progression of cholestatic liver disease observed in PFIC3 mutations found instead in the *Mdr2*^−/−^ mice [138].

Accordingly, hydrophilic manipulation of bile acid composition via the introduction of THBAs could prevent the progression of cholestatic liver injury in PFIC3 patients.

In conclusion, all the in vivo and in vitro scientific evidence reported above points to the potential therapeutic implications of the diet and of the manipulation of the microbiota via prebiotics, probiotics and non-resorbable antibiotics in order to reduce the risk of HBCs in patients suffering from inherited cholestatic diseases.

## 6. Discussion

Liver cancer has historically been associated with the classic forms of PFIC type 2 and 3, characterized by advanced fibrosis and cirrhosis, which are conditions predisposing to HBCs. New technologies have made it possible to discover new genes responsible for inherited cholestatic diseases in the most recent years and a risk of HBCs associated with non-progressive forms of cholestasis and heterozygous conditions could be shown. Many proteins are involved in the synthesis, transport and metabolism of BAs in hepatocytes, biliary canaliculus and gut cells. The result of stopping one or more levels of this complex process is an increase in serum and liver cell levels of BAs. BAs are steroid-based molecules synthesized from cholesterol in the liver and involved in glucose and lipid metabolism as well as in the absorption of dietary fats and fat-soluble vitamins. Since BAs regulate main inflammatory pathways and gut microbiota balance, it is not surprising that BAs are involved in the development of certain gastrointestinal cancers [2].

Normal homeostasis of BAs is essential to avoid liver inflammation and to reduce the risk of fibrosis; high levels of BAs induced HCC and CCA via metabolic disorders, hepatic inflammation, oxidative stress, fibrosis, resistance to apoptosis, hyperproliferation and via their cytolytic effects in cholestatic diseases as well as PFIC and Alagille syndrome [2]. FXR and Notch pathways are among the most studied ones there are. Notch signalling is involved in many aspects of the development of the biliary tree, tissue repair and liver carcinogenesis. Defects in Notch signalling impairs the ability of liver repair against injury, while excessive activation may be involved in liver cancer. Notch receptor activation releases NICD that migrates into the nucleus. Here, NICD interacts with RBPJκ able to promote the transcription of Notch target genes [6]. Interestingly, a target gene upregulated by NOTCH signalling is *HNF1B*, having a vital role in the differentiation of hepatoblasts into ductal plate cells and the inclusion of the developing duct into the portal space. Moreover, *HNF1B* downregulation and mutations in *HNF1B* have been associated with neonatal or late-onset cholestasis and with common cancers, including endometrial, prostate, ovarian, hepatocellular, renal and colorectal tumours [139]. FXR is one of the most studied BAs receptors regulating the BAs synthesis, metabolism and intestinal reuptake.

After activation, FXR inhibits CYP7A1 expression, stimulates FGF-19 to stop CYP7A1 and CYP8B1 synthesis through the fibroblast growth factor receptor 4 (FGFR4) pathway in the hepatocytes, downregulates NTCP repressing the uptake of BAs by the liver and finally increases the expression of OSTa/b, involved in BAs excretion from the liver to the portal vein and in their intestinal reuptake. Interestingly, FXR is directly involved in PFIC4 and indirectly in PFIC2 and PFIC3. Its malfunction leads to the blocking of BAs efflux from the liver to the canalicular lumen through the downstream of BSEP and MDR3, thereby exposing hepatocytes to the high cytotoxicity of BAs [80]. FXR up-regulates the FGF15/19-FGFR4 signalling, which may increase the risk of HCC [140,141]. Obeticholic acid (OCA), is the most active physiological ligand for FXR and it was approved by the U.S. FDA in 2016 as a second-line treatment for primary biliary cholangitis [142]. Its potential role in preventing the risk of HBCs has yet to be demonstrated.

The exponential progress in the knowledge of the mechanisms underlying bile salt homeostasis and the discovery of new genes and new proteins involved in such processes both in the liver and intestine has enabled the development of new drugs such as odevixibat (Bylvay™). This is in fact a small molecule inhibitor of the apical sodium-dependent bile acid transporter (ASBT), also known as ileal bile acid transporter (IBAT), the first drug approved in the EU for the treatment of PFIC while is used worldwide also in Alagille syndrome and biliary atresia [143]. The BAs concentrations are crucial in determining the progression of fibrosis and the risk of HCC. Therefore, the role of biliary diversion (BD) is not surprising in increasing native liver survival in patients with BSEP deficiency and mild to moderate phenomena [67]. Although not significant, there was a trend toward less development of HCC in patients with a BD in a multicentre, retrospective cohort study including 264 patients: HCC occurred in 2/61 (3%) patients that underwent BD and in 13/180 (7%) that did not (*p* = 0.32). Therefore, drugs such as FXR agonists or IBAT Inhibitors could reduce the risk of HBCs development in subjects suffering from inherited cholestatic diseases by lowering serum and intracellular levels of BAs. The well-known association of cholestatic diseases such as primary sclerosing cholangitis with inflammatory intestinal disorders and biliary and intestinal cancers highlights the role of the gut microbiome-mediated modulation of bile acids.

The liver receives gut microbiome metabolites via the portal vein: antigens, BAs, lipopolysaccharide, choline, indole derivatives, and short-chain fatty acids are involved in the carcinogenesis process, in downregulation of the liver immune system, in the progression of many liver diseases, including nonalcoholic fatty liver disease [144].

While the liver synthesizes primary BAs, the microbiota produces secondary metabolites through bacterial bile salt hydrolases. This enzyme catalyzes the hydrolysis of conjugated BAs to deconjugated BAs and amino acids.

Deoxycholic acid is a secondary BA involved in the production of interleukin-6, growth-regulated oncogene-alpha, chemokine (C-X-C motif) ligand (CXCL) 9, and prostaglandin E2 (PGE2) by hepatic stellate cells.

This group of elements have several inflammatory and prooncogenic properties, leading to HBCs development [140].

Furthermore, secondary BAs, such as ω-muricholic acid, favourite carcinogenesis via downregulation of immunosurveillance, reducing the accumulation of NKT cells in the liver [145]. Furthermore, the diet regulates the gut microbial composition: in *Tlr5*-deficient mice with dysbiosis, a diet enriched with soluble fibres, such as inulin, pectin and fructooligosaccharides, was able to induce cholestatic liver cancer. There is a clear association between the gut microbiome, secondary BAs, immune system, diet and liver cancer. Therefore, the manipulation of the microbiota could reduce the risk of tumours in subjects with high levels of BAs due to inherited cholestatic diseases through the reduced production of secondary metabolites.

In this setting, the use of vancomycin therapy depletes proinflammatory bacterias as well as *Clostridium*, increases liver NKT cell accumulation and reduces liver cancer development [133]. On the other hand, vancomycin administration has been occasionally associated with improving primary sclerosing cholangitis patients although evidence of efficacy has not yet been provided [146]. Other antibiotics, namely rifaximin and norfloxacin, were demonstrated to be useful in the treatment of human liver cancer too, as well as some probiotics such as *Bifidobacterium* that may inhibit cancer growth in mice models by increasing the production of anti-inflammatory metabolites [144].

In conclusion, the genes responsible for hereditary cholestatic diseases such as PFIC and Alagille syndrome play a key role in determining BAs’ serum and intrahepatic levels. Identifying subjects at risk of developing HBCs could allow adequate surveillance strategies and personalized therapeutic interventions through drugs capable of acting on BAs’ synthesis, transport and recirculation and the microbiota responsible for their metabolism in substances favouring carcinogenesis.

## 7. Conclusions

In conclusion, our review underlines the association between familial intrahepatic cholestasis and HBCs.

The fast advances in diagnostic genetic methods and NGS, WES and WGS, have helped us understand the relationship between a broad spectrum of cholestatic disorders and the development of HBCs. In particular non-progressive forms of inherited cholestatic disorders, similarly to conditions produced by heterozygous or compound mutations in PFIC genes, were shown to be associated with the onset of HBCs in otherwise apparently healthy livers or with modest biochemical abnormalities such as an isolated increase in GGT. BAs levels and metabolites may be used to identify individuals at high risk of HCC development. Consequently, subjects with risk factors for PFIC such as a personal or familial history of DIC, ICP, LPAC, HBCs and cryptogenic cholestasis abnormalities may benefit from a screening measurement of BAs.

Patients with elevated BAs, in addition to genetic tests, could be offered ultrasound surveillance programs and diet correction interventions, especially if metabolic risk factors are concurrently present. Subjects with pathogenic, probably pathogenic mutations or mutations of uncertain significance according to the American College of Medical Genetics and Genomics standards [147] could also be considered for pharmacological treatments to reduce serum levels of BAs and even possibly through the modification of the gut microbiota aiming at primary prevention against the occurrence of familial cholestasis-related HBCs.

However, future studies are needed to understand better the role of inherited cholestatic liver diseases, the microbiota’s dysbiosis and the immune response in the genesis of HBCs. In the era of multikinase inhibitors and immune checkpoint inhibitors, new research oriented on the correction of the deficient proteins involved in the synthesis, transport and reuptake of BA in the liver and the gut, on faecal microbiota transplantation and antibiotics/probiotics use in special populations, are amazing opportunities in primary, secondary prevention and the personalized treatment of liver cancers.

## Figures and Tables

**Table 1:** List of genes associated with progressive familial intrahepatic cholestasis and their related phenotypes.

Year	Gene	Protein	Phenotypes	Hepatobiliary Cancers
1998	ATP8B1	ATP8B1	ICP BRIC PFIC 1	Not reported
1998	ABCB11	BSEP	ICP DIC LPAC BRIC PFIC 2	HCC CCA
1996	ABCB4	MDR3	ICP DIC LPAC PFIC 3	HCC CCA Gallbladder cancer
2014	TJP2	TJP2	ICP PFIC 4	HCC CCA
2016	NR1H4	FXR	ICP PFIC 5	Not reported
2017	MYO5B	MYO5B	BRIC MVID MYO5B-PFIC	Not reported
2019	USP53	USP53 protein	BRIC PFIC 7	Not reported
2019	KIF12	KIF12	PFIC 8	Not reported
2020	SLC51A	OSTα-OSTβ	PFIC 6	Not reported

Abbreviations: BRIC, benign recurrent intrahepatic cholestasis; PFIC, progressive familial intrahepatic cholestasis; ICP, intrahepatic cholestasis of pregnancy; HCC−CCA, hepatocellular carcinoma-cholangiocarcinoma; DIC, drug-induced cholestasis; LPAC, low-phospholipid-associated cholelithiasis.

**Table 2:** Cases described in PubMed of HBCs associated with mutations in cholestasis-related genes.

Patient †/Gender/Origins	Age, PFIC Onset	Age, HBC Type and Liver Histology	Gene	Nucleotide Changes	Predicted Consequences	PMID
A/M/NEC	Cho from 3 wk	21 mo (incidental in explant; AFP 199 ng), at LT	*ABCB11*	c.1939delA/c.2012-8T>G	p.G648Vfs*6/splice site disruption	16871584
B/F/NEC	Cho from 2 wk, hospitalized for evaluation aged 12 wk	28 mo, at open biopsy; AFP not determined	*ABCB11*	c.2178 + 1G>A/c.74C>A	Splice site disruption/p.S25*	16871584
C/M/NEC	Cho from birth	23 mo (AFP 30k ng; liver mass); histologic diagnosis at necropsy, 24 mo	*ABCB11*	c.1445A>G/c.3691C>T	p.D482G/p.R1231W	16871584
D/M/NEC	Cho from 3 wk	22 mo (AFP 158k ng); liver mass; lung and bone lesions; chemotherapy given; histologic diagnosis at LT, 25 mo	*ABCB11*	c.890A>G/c.890A>G	p.E297G/p.E297G	16871584
E/M/NEC	Growth failure from 6 mo; diagnosed 9.5 mo	29 mo (incidental in explant; AFP 6.4k ng), at LT	*ABCB11*	c.611 + 1G>A/c.890A>G	Splice site disruption/p.E297G	16871584
F/M/NEC	Cho from 6 wk	16 mo (clinically unsuspected), at necropsy; AFP not determined	*ABCB11*	c.908 + 1G>A/not known	Splice site disruption/not known	16871584
G/F/A	Cho from 6 wk	15 mo, HCC (AFP 11k ng); histologic diagnosis at LT, 16 mo	*ABCB11*	c.1416T>A/c.1416T>A	p.Y472*/p.Y472*	16871584
H/M/NEC	Evaluation at 6 mo for J and growth failure	52 mo (marked increase in abdominal size; tumour metastasized at diagnosis; AFP 2 × 106 ng), at open biopsy	*ABCB11*	c.890A>G/IVS13del-13ˆ-8	p.E297G/splice site disruption	16871584
I/M/CAC	Cho from birth	13 mo (incidental in explant; AFP 831 ng), at LT	*ABCB11*	c.2343 + 2T>C/c.2343 + 2T>C	Splice site disruption	16871584
J/M/CAC	Cho from 1 wk	14 mo (AFP 4k ng; liver mass), at biopsy; confirmed at LT, 15 mo	*ABCB11*	c.2316T>G/c.2316T>G	p.Y772*/p.Y772*	16871584
K/M/NEC	Cho from 3 mo	26 mo; HCC metastasized at diagnosis (AFP not reported); at biopsy	*ABCB11*	None sought	None predicted	16871584
A/F/Hi	2 mo	Giant cell hepatitis and mild portal-tract fibrosis, biliary Cir (3 years), CCA at 4/6/12 yrs	*ABCB11*	c.1723C>T/12.5 Mb del	p.R575*/12.5 Mb del	17452236
B/F/C	J and I in infancy	Giant cell hepatitis (2 mo), hepatic resection revealed advanced biliary Cir with left-lobe peripheral CCA	*ABCB11*	c.890A>G/c.2343 + 1G>T	p.Q297G/splice site disruption	17452236
1/-/CA-A *			*ABCB11*	c.379delA/c.379delA	p.T127Hfs*6/p.T127Hfs*6	18395098
7/-/CA-A *			*ABCB11*	c.3213 + 1delG/c.3213 + 1delG	Splice defect	18395098
65			*ABCB11*	c.3382C>T/c.3382C>T	p.R1128C/p.R1128C	18395098
45a/-/EU			*ABCB11*	c.1238T>G/c.1238T>G	p.L413W/p.L413W	18395098
45b/-/EU			*ABCB11*	c.1238T>G/c.1238T>G	p.L413W/p.L413W	18395098
47a/-/EU			*ABCB11*	c.149T>C/c.149T>C	p.L50S/p.L50S	18395098
47b/-/EU			*ABCB11*	c.149T>C/c.149T>C	p.L50S/p.L50S	18395098
83/-/EU			*ABCB11*	c.937C>A/c.1445A>G	p.R313S/p.D482G	18395098
105/-/EU			*ABCB11*	c.1445A>G/not identified	p.D482G/not identified	18395098
5a/-/-	10 mo	4 yrs; H, S, I, J, LF; trifocal HCC, AFP 931 ng	*ABCB11*	Not reported	p.Y354*/p.G982R	20232290
16/-/-	1 mo	H, I, recurrent J, LF, AFP 1500 ng (17 mo)	*ABCB11*	Not reported	p.R1231W/p.I528*	20232290
24/-/-	1 mo	H, S, permanent J, I, bifocal HCC, normal values of AFP (10 yrs), LF (12 yrs)	*ABCB11*	Not reported	p.R1153C/c.3213 + 4A>G	20232290
27/-/-	3 wk	H, I, decreased J, AFP 5000 ng (10 yrs), HCC (1 nodule resected)	*ABCB11*	Not reported	p.G982R/p.R1001R (predicted to affect splicing)	20232290
32/-/-	1 mo	H, pemanent J, I, HCC (2 nodules), AFP 3600 ng, LF (7 mo)	*ABCB11*	Not reported	p.R698H/nf	20232290
33/-/-	1 wk	H, permanent J, DS, I AFP 124752 (2mo) and 19770 ng (5mo), no nodule, LF (4 mo)	*ABCB11*	Not reported	p.R698H/nf	20232290
1/F/-	P at the age of 8 mo	8 mo	*ABCB11*	Not reported	p.A389P/p.R1226H	25016225
10/F/EU	54 yrs	Periportal fibrosis, mild ductular reaction, steatosis, biliary dysplasia, CCA	*ABCB4*	c.1005 + 5G>A/wt	Splicing/wt	22331132
13/F/EU	55 yrs	Biliary Cir, macronodule with well-differentiated HCC	*ABCB4*	c.959C>T/wt	p.S320F/wt	22331132
GWAS Icelandic population	<40 yrs	Liver, gallbladder and gallways cancer OR 2.42	*ABCB4*	c.1865G>A	p.G622E	25807286
GWAS Icelandic population	<40 yrs	Liver, gallbladder and gallways cancer OR 3.07	*ABCB4*	c.1333_1334delCT	p.L445Gfs*22	25807286
GWAS Icelandic population	<40 yrs	Liver, gallbladder and gallways cancer OR 4.75	*ABCB4*	c.1529A>G	p.N510S	25807286
GWAS Icelandic population	<40 yrs	Liver, gallbladder and gallways cancer OR 0.99	*ABCB4*	c.711A>T	p.I237=	25807286
1/-/EU	LPAC	CCA diagnosed	*ABCB4*	c.1405A>T/wt	p.R469W/wt	32893960
2/-/EU	LPAC	CCA diagnosed	*ABCB4*	c.1268A>C/wt	p.Q423P/wt	32893960
3/-/EU	LPAC	HCC diagnosed	*ABCB4*	c.760G>A/wt; c.1546A>G/wt; c.2363G>A/wt	p.A254T/wt; p.M516V/wt; p.R788Q/wt	32893960
1/F/C	Neonatal onset, 26 mo	At liver biopsy moderately differentiated HCC in a chronic Cho with Cir	*TJP2*	c.2668-1G>T/c.2438dupT	Splice defect/p.N814Qfs*28	25921221
2/M/C	Neonatal onset, 6 mo	Liver biopsy at 6 mo showed C, giant cell transformation, and micronodular Cir	*TJP2*	c.817delG/c.817delG	p.A273Pfs*38/p.A273Pfs*38	25921221
1/F/-	1 month	7 yrs	*TJP2* NM_001170416.1	c.(2659 + 1_2660-1)_(2760 + 1_2761-1)del/c.(2659 + 1_2660-1)_(2760 + 1_2761-1)del	Skipping exon 18/skipping exon 18	28733884
P2.5/M/-	20 yrs	23 yrs	*TJP2*	c.3334C>T/c.3334C>T	p.Q1112*/p.Q1112*	32089630
1/M/-	ALGS at 13 wk of age	3 yrs	*JAG1*	De novo mutation of the protein-encoding JAG1-region of chr. 20 (not shown by the authors)		20714715
-/F/-	ALGS around age 35 yrs		*JAG1*	c.693_694del state not specified	p.Arg231Serfs*10	33369123
1/-/-	Cir, 1.5 y		*JAG1*	c.551G>A/wt	p.R184H/wt	32180165
2/-/-	Cir, 2 yrs		*NOTCH2*	c.5830G>A/c.5830G>A	p.G1944S/p.G1944S	32180165
1/F/Ja	CTLDN2; 40 yrs, after her first baby	40 yrs	*SLC25A13*	c.1180 + 1G>A/c.1180 + 1G>A	Splice site disruption	14606711
1/M/-	No symptoms	50 yrs, HCC and intrahepatic CCA	*SLC25A13*	c.1180 + 1G>A/c.1180 + 1G>A	Splice site disruption	18385606
1/M/T	CTLDN2; 34 yrs	HCC at 48 yrs	*SLC25A13*	c.852_855del/c.852_855del	p.M285Pfs*2/p.M285Pfs*2	17000460
1/M/Ch	After birth	HCC at 6 yrs	*SLC25A13*	c.852_855del/c.852_855del	p.M285Pfs*2/p.M285Pfs*2	30591617
1/M/-	After birth	HCC at 16 mo	*HNF1B*	1.5 Mb deletion of chr. 17/wt	No protein/wt	29727438

† The first letters and numbers shown in the column “Patient/Gender/Origins” are the same as in the original papers to identify patients; * The symbol indicates acknowledged parental consanguinity. Origins abbreviations: A, Arabic; C, Caucasian; CA, Central Asian; CAC, Central Asian Caucasian; Ch, Chinese; Hi, Hispanic; Ja, Japanese; NEC, Northern European Caucasian; SA, South Asian; T, Taiwanese. Other abbreviations: AFP, alpha-phetoprotein; BD, biliary diversion; Cho, cholestasis, CCA, cholangiocarcinoma; Cir, cirrhosis; CTLDN2, adult-onset type II citrullinemia; del, deletion; GWAS, genome-wide association study; H, hepatomegaly; HBC, hepatobiliary cancer; HCC, hepatocellular carcinoma; I, itching; J, jaundice; LF, liver failure; LPAC, low-phospholipid-associated cholelithiasis; LT, liver transplantation; MB, megabases; mo, months; nf, not found; ng, nanograms/mL; NLT, normalized liver tests; OR, odd ratio; PFIC, progressive familial intrahepatic cholestasis; PMID, PubMed-Indexed for MEDLINE; wk, weeks; wt; wild type; y, year; yrs, years.

## Abbreviations

A	Arabic
AFP	alpha-phetoprotein
ARE	apical recycling endosome
ASBT	apical sodium-dependent bile acid transporter
AP	amino-phospholipids
BAs	bile acids
BD	biliary diversion
BSEP	bile salt export pump protein
BRIC	benign recurrent intrahepatic cholestasis
C	Caucasian
CA	Central Asian
CAC	Central Asian Caucasian
CCA	cholangiocarcinoma
Ch	Chinese
Cho	cholestasis
Cir	cirrhosis
CFTR	cystic fibrosis transmembrane conductance regulator
CNVs	copy number variants
CTLDN2	adult-onset type II citrullinemia
CYP7A1	cholesterol 7α-monooxygenase
del	deletion
DIC	drug-induced cholestasis
DILI	drug-induced liver injury
DSBs	double-strand breaks
ER	endoplasmic reticulum
FGF19	fibroblast growth factor 19
FGFR14	fibroblast growth factor receptor 4
FIC	familial intrahepatic cholestasis
FIC 1	familial intrahepatic cholestasis deficiency type 1 protein
FXR	farnesoid X receptor
FTTDCD	failure to thrive and dyslipidemia caused by citrin deficiency
GGT	gamma-glutamyl transferase
GWAS	genome-wide association study
H	hepatomegaly
Hi	Hispanic
HBCs	hepatobiliary cancers
HCC	hepatocellular carcinoma
HNF-1B	hepatocyte nuclear factor-1beta
I	itching
IBAT	ileal bile acid transporter
ICP	intrahepatic cholestasis of pregnancy
INDELs	DNA insertions and deletions
J	jaundice
Ja	Japanese
JAG-2	jagged canonical notch ligand-2
KIF12	kinesin family member 12
LF	liver failure
LT	liver transplantation
MODY	maturity-onset diabetes of the young
MVID	microvillus inclusion disease
LPAC	low-phospholipid-associated cholelithiasis
LT	liver transplantation
MB	megabases
MDR	multidrug resistance protein
mo	months
MRP	multidrug resistance protein
NEC	Northern European Caucasian
NGS	next-generation sequencing
mo	months
MDR	multidrug resistance protein
ng	nanograms/mL
NKT	natural killer T
nf	not found
NICD	notch intracellular domain
NLT	normalized liver tests
NOTCH-1,2	notch homolog-1,2 translocation-associated
NTCP	sodium taurocholate cotransporting polypeptide
OMIM	Online Mendelian Inheritance in Man
OR	odd ratio
OATP	organic anion transporting polypeptide
OST α/β	organic solute transporter alpha/beta
PC	phosphatidylcholine
PE	phosphatidylethanolamine
PELD PFIC	paediatric end-stage liver disease progressive familial intrahepatic cholestasis
PGS	panel gene sequencing
PMID	PubMed-Indexed for MEDLINE
PS	phosphatidylserine
RBPjk	recombining binding protein suppressor of hairless
RCAD	renal cysts and diabetes syndrome
ROS	reactive oxygen species
SA	South Asian
SL25A13	solute carrier family 25 member 13
STRs	short tandem repeats
SVs	structural variants
T	Taiwanese
TGR5	G-protein-coupled bile acid receptor
TJP2	tight junction protein 2 gene
THBA	tetrahydroxylated bile acids
UDCA	ursodeoxycholic acid
USP53	ubiquitin-specific peptidase 53
VIPAR	VPS33B interacting protein, apical–basal polarity regulator
VPS33B	vacuolar protein sorting associated protein 33B
WES	whole-exome sequencing
WGS	whole-genome sequencing
wk	weeks
wt	wild type
y	year
yrs	years
